# The Prognostic Significance of Low-Triiodothyronine Syndrome in Aneurysmal Subarachnoid Hemorrhage

**DOI:** 10.3390/biomedicines14030603

**Published:** 2026-03-09

**Authors:** Adrianna Lebiedzińska, Małgorzata Burzyńska, Jowita Woźniak, Waldemar Goździk

**Affiliations:** 1 Clinical Department of Anesthesiology and Intensive Therapy, University Clinical Hospital in Wroclaw, 50-556 Wroclaw, Poland; 2 Clinical Department of Anesthesiology and Intensive Therapy, Faculty of Medicine, Wroclaw Medical University, 50-367 Wroclaw, Poland; malgorzata.burzynska@umw.edu.pl (M.B.); waldemar.gozdzik@umw.edu.pl (W.G.); 3 Clinical Department of Neurosurgery, University Centre for Neurology and Neurosurgery, University Clinical Hospital in Wroclaw, 50-556 Wroclaw, Poland; jowita.wozniak@usk.wroc.pl

**Keywords:** subarachnoid hemorrhage, low-triiodothyronine syndrome, non-thyroidal illness syndrome, thyroid hormones, biomarkers, critical care, mortality

## Abstract

**Background:** Aneurysmal subarachnoid hemorrhage (aSAH) is associated with high early mortality and long-term disability. Prognostic assessment relies mainly on neurological grading scales, which may incompletely capture the systemic metabolic response to acute brain injury. Non-thyroidal illness syndrome (NTIS), particularly low triiodothyronine syndrome (LT3S), is common in critical illness, but its prognostic relevance in aSAH remains unclear. **Objectives:** To evaluate the prognostic impact of early thyroid hormone alterations on 30-day mortality and early clinical outcomes including delayed cerebral ischemia (DCI) in patients with aSAH, with particular emphasis on the magnitude of triiodothyronine (T3) deficiency. **Methods:** We conducted a retrospective single-center observational cohort study of 157 consecutive adult patients admitted with confirmed aSAH between 2014 and 2025. Serum free triiodothyronine (fT3), free thyroxine (fT4), and thyroid-stimulating hormone (TSH) were measured within 72 h of admission. Hormone values were normalized to contemporaneous reference intervals to generate continuous reference-adjusted metrics (FT3_level, TSH_level). Associations with 30-day in-hospital mortality were analyzed using logistic regression and Cox proportional hazards models adjusted for admission variables including age, sex, APACHE II score, World Federation of Neurosurgical Societies grade, Fisher grade, and treatment modality. **Results:** Binary LT3S classification was frequent but not independently associated with 30-day mortality. In contrast, lower FT3_level values were significantly associated with increased mortality and shorter survival time. In logistic regression analyses, each 0.1 increase in FT3_level was associated with an 18% lower odds of death (adjusted OR 0.82, 95% CI 0.69–0.97). This association persisted after adjustment for established clinical severity measures and was concordant with time-to-event analyses. FT3_level was not correlated with TSH_level, consistent with NTIS. Endovascular coiling was associated with more pronounced peripheral fT3 deficiency (*p* < 0.05) but was not independently associated with mortality. FT3_level was not independently associated with early neurological status or functional outcome at hospital discharge. **Conclusions:** Lower FT3_level values were independently associated with higher 30-day mortality, indicating that early peripheral T3 reduction reflects clinically relevant metabolic vulnerability in aSAH.

## 1. Introduction

### 1.1. Clinical Burden and Prognostic Challenges

Aneurysmal subarachnoid hemorrhage (aSAH) is among the most devastating cerebrovascular events, accounting for approximately 5% of all strokes and affecting 6 to 9 individuals per 100,000 annually in developed countries [1,2]. Despite advances in neurocritical care, early mortality remains substantial, ranging from 30% to 40% [2,3,4], and up to one third of survivors experience persistent neurological deficits and long-term disability [5]. Consequently, identifying early and reliable prognostic markers beyond conventional neurological grading systems remains a major clinical priority [6].

Traditional prognostication in aSAH relies primarily on clinical and radiological severity scales [3,6]. Although these tools effectively capture neurological injury and global physiological derangement, they may not fully reflect the systemic metabolic and endocrine response to acute brain injury [7]. Aneurysmal SAH is accompanied by a profound stress response involving inflammatory activation and endocrine dysregulation [7,8], extending beyond focal neuronal damage and influencing early outcomes [9,10]. Among that, delayed cerebral ischemia (DCI) represents one of the most serious and prognostically relevant events, strongly associated with neurological deterioration, prolonged stay in intensive care unit (ICU), and poor functional outcome [6,7,8,9,10].

### 1.2. Thyroid Hormone Alterations in Critical Illness

Non-thyroidal illness syndrome (NTIS) represents a common endocrine response to acute systemic illness, occurring in the absence of intrinsic thyroid disease and reflecting a complex interplay between adaptive and maladaptive mechanisms [11,12]. The hallmark biochemical feature of NTIS is impaired peripheral conversion of thyroxine (T4) to triiodothyronine (T3), accompanied by increased production of biologically inactive reverse T3 [13,14], largely driven by illness-related alterations in iodothyronine deiodinase activity [13]. In parallel, central regulation of the hypothalamic–pituitary–thyroid axis is disrupted, with thyroid-stimulating hormone (TSH) levels typically remaining normal in early stages of illness and becoming suppressed as disease severity progresses [11].

A key clinical manifestation of NTIS is low-triiodothyronine syndrome (LT3S), defined by reduced circulating free T3 (fT3) concentrations in the presence of normal or suppressed TSH levels [11,13].

LT3S has been reported across diverse critical care populations [15,16,17] and has been associated with adverse outcomes [18,19], including increased mortality in sepsis [20,21], respiratory failure [22], neurological and neurosurgical cohorts [23,24]. However, whether reduced fT3 levels represent merely a marker of global illness severity or provide independent prognostic information in patients with aSAH remains unclear.

In aSAH, the hypothalamic–pituitary axis and peripheral thyroid hormone metabolism are particularly susceptible to disruption. Exposure to subarachnoid blood products, intracranial hypertension, cerebral hypoperfusion, inflammatory cascades, catecholamine surges, and pharmacological interventions may all contribute to early thyroid hormone alterations [7,8,9,10]. Previous endocrine studies in aSAH have largely focused on descriptive hormonal changes, with limited assessment of prognostic relevance and frequent reliance on raw hormone concentrations or binary LT3S classifications [7,8,9,24,25], often without multivariable adjustment for established severity markers or evaluation of dose–response relationships [24,25]. Consequently, it remains unclear whether quantitative measures of early fT3 deficiency provide independent prognostic information beyond conventional neurological and systemic risk stratification. Advances in neurocritical care and biomarker analytics have renewed interest in systemic metabolic responses as potential contributors to outcome variability in aSAH.

### 1.3. Rationale for Quantitative fT3 Assessment

Although the association between low fT3 and poor outcomes in critical illness is well recognized [26,27,28], the optimal approach to quantifying fT3 deficiency and its prognostic relevance remains uncertain. Binary LT3S classification reduces a biologically continuous phenomenon to a dichotomous variable, potentially limiting statistical power and obscuring dose–response relationships [28,29]. Determining whether early fT3 measurements provide prognostic information beyond established severity scores—and whether standardized, reference-adjusted metrics improve risk stratification—is essential for evaluating whether endocrine markers may provide complementary prognostic information alongside established clinical scales [22,23].

### 1.4. Objectives

The objectives of this study were to characterize early thyroid hormone alterations and the prevalence of LT3S in patients with aSAH, to evaluate the independent and combined effects of quantitative fT3 deficiency on 30-day in-hospital mortality, to assess their associations with early neurological and functional outcomes, including DCI, and to compare the prognostic performance of binary LT3S classification with continuous, reference-range–adjusted fT3 metrics.

## 2. Materials and Methods

### 2.1. Study Design and Population

This retrospective single-center observational cohort study initially included 249 consecutive adult patients admitted with SAH and treated in the Department of Anesthesiology and Intensive Care of the University Clinical Hospital between July 2014 and July 2025. Diagnosis was established using cranial computed tomography (CT), computed tomography angiography (CTA), or digital subtraction angiography (DSA). Patients were treated according to a standardized institutional protocol based on American Heart Association/American Stroke Association guidelines.

Inclusion criteria were: (1) age ≥ 18 years; (2) hospital admission within 24 h of symptom onset; and (3) surgical or endovascular aneurysm occlusion within 48 h.

Exclusion criteria included: (1) non-aneurysmal SAH; (2) pre-existing central nervous system disease; (3) known thyroid or pituitary disorders; (4) immunosuppressive therapy; (5) intraoperative complications; (6) signs of irreversible brain injury at admission (including bilateral fixed pupil dilation); (7) pregnancy; and (8) incomplete clinical or laboratory data.

**Figure 1:**
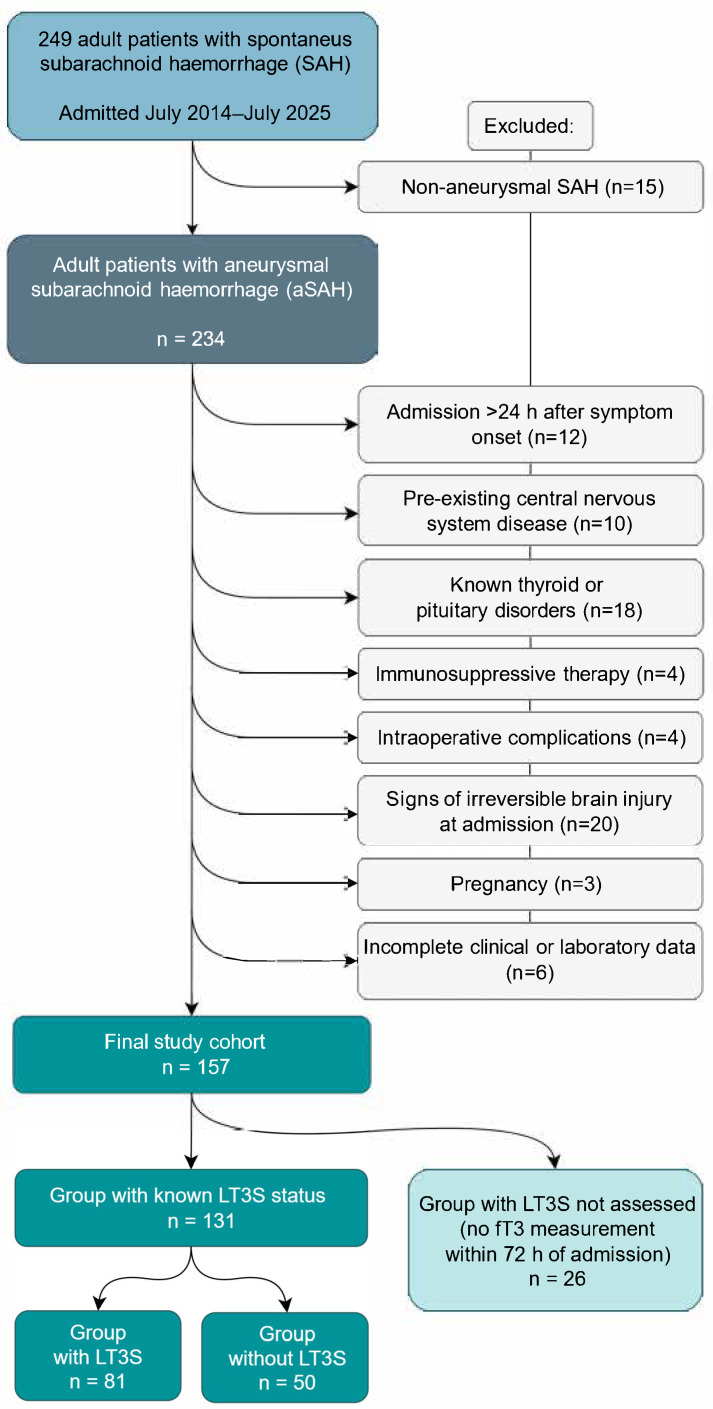
Flowchart of patient selection and study cohort formation.

Patient selection and reasons for exclusion are summarized in Figure 1. After application of exclusion criteria, 157 patients constituted the final study cohort and were included in the analysis (Table 1). To assess potential selection bias related to missing hormone measurements, we compared patients with and without available fT3 values.

### 2.2. Thyroid Hormone Assessment

Serum concentrations of fT3, fT4, and TSH were measured within the first 72 h after admission as part of routine clinical evaluation. This time window was selected intentionally to correspond to the phase of early brain injury (EBI) following aSAH, typically defined as the first 72 h after hemorrhage, during which the most profound neuroendocrine, inflammatory, and metabolic disturbances occur [6,7]. The aim of the measurement was therefore to characterize the early systemic response to acute brain injury rather than to obtain a stable premorbid endocrine baseline. LT3S was defined as an fT3 concentration below the contemporaneous laboratory-specific reference range in the presence of normal or suppressed TSH, in patients without clinical or laboratory evidence of primary thyroid disease. Patients without available fT3 measurements were classified as “LT3S not assessed” and excluded from analyses requiring thyroid status stratification. Missing fT3 measurements reflected the pragmatic nature of retrospective data collection. No imputation was performed, and analyses were conducted as a complete-case analysis. Measurements reflected routine clinical laboratory workflow, meaning that sampling was not protocolized to an exact hour but remained anchored within the defined EBI period. This pragmatic approach was intended to preserve real-world external validity of early-phase biomarker assessment in neurocritical care. Because missingness was not associated with baseline severity or adjusted mortality risk, additional sensitivity analyses were not considered likely to materially change the results.

### 2.3. Reference-Range–Adjusted Thyroid Hormone Metrics

As emphasized in methodological guidance for clinical data harmonization, the interpretation of laboratory results depends on assay-specific reference ranges, which may differ between instruments, laboratories, and calendar periods, rendering unadjusted measurements non-comparable [30]. Although laboratory reference ranges for thyroid hormones varied over the study period due to changes in assay calibration, measurement units remained constant throughout the study (fT3 in pg/mL, fT4 in ng/dL, and TSH in µIU/mL). Consequently, identical numerical fT3 values could be interpreted differently depending on the contemporaneous laboratory reference interval. Direct comparison of raw fT3 concentrations across time would therefore introduce systematic misclassification unrelated to biological differences and was not considered methodologically appropriate.

The contemporaneous assay-specific reference interval was defined by its lower reference limit (LRL) and upper reference limit (URL). These parameters were used to anchor the min–max transformation for each individual measurement. To mitigate the impact of inter-assay variability and enable comparability across changing laboratory reference intervals, fT3 concentrations were normalized relative to the assay-specific reference range using a min–max transformation (linear transformation anchored to LRL and URL). As a monotonic transformation, these rescaling preserves rank ordering and effect directions while improving comparability across assays without implying analytical equivalence between measurement platforms. The primary normalized metric, termed FT3_level, was calculated as:

(1) $$FT3_{level}=\frac{fT3-LRL}{URL-LRL}$$

Because the transformation is monotonic, all rank relationships between observations are preserved; therefore, regression coefficients and hazard ratios derived from FT3_level reflect the same biological gradients as those based on raw fT3 values, while enabling comparability across assays.

**Figure 2:**
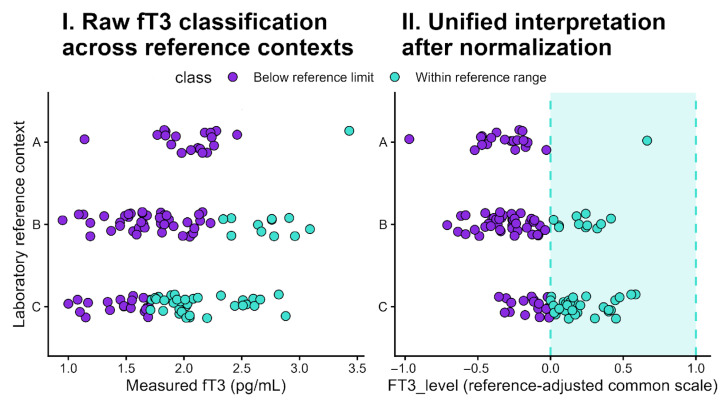
Impact of assay-specific reference ranges on fT3 interpretation and effect of reference-adjusted normalization. **Panel I** shows raw fT3 values stratified by three laboratory reference contexts (A–C). Identical numerical concentrations are classified differently depending on the contemporaneous reference interval (below vs. within range). **Panel II** presents the same measurements after transformation to the common FT3_level scale, where 0–1 represents the assay-specific reference range. Normalization aligns patients from different assays onto a unified clinical continuum and removes artificial between-assay discrepancies. Colors indicate laboratory classification: purple—below reference limit; turquoise—within reference range.

This approach expresses each result as its relative position within the applicable reference interval, where values < 0 indicate biochemical deficiency, 0–1 represent physiological concentrations, and >1 reflect supraphysiological levels (see Figure 2 for graphical rationale). For example, an FT3_level of 0.5 indicates that the measured fT3 lies halfway within the normal range, an FT3_level of 0.25 corresponds to the first quartile of the reference interval, whereas an FT3_level of −0.5 indicates a concentration located half of the reference interval below the lower reference limit.

The rationale for this transformation is consistent with previous evidence demonstrating that the position of fT3 within the individual reference range carries greater prognostic information than the absolute value alone [29]. Similar normalization strategies are widely used to enable comparison of laboratory parameters measured with different assays and reference limits [29,30].

An analogous metric was constructed for TSH (TSH_level), using the contemporaneous laboratory reference limits.

(2) $${TSH}_{level}=\frac{TSH-LRL}{URL-LRL}$$

In this formulation, values below zero indicate deficits in hormone concentrations, values between zero and one correspond to physiological concentrations within the reference range, and values above one reflect supraphysiological levels.

To improve both longitudinal robustness and clinical interpretability, fT3 deficiency was modeled using two complementary continuous measures: a reference-range–adjusted metric (FT3_level) and the raw fT3 deficit (expressed in pg/mL). Raw fT3 deficit was calculated as the difference between the LRL and the measured fT3 concentration:

(3) raw fT3 deficit=LRL−fT3

Positive values denote a concentration below the LRL, whereas negative values indicate fT3 concentrations within or above the reference range.

### 2.4. Statistical Analysis

The association between missing fT3 measurements and mortality was explored using adjusted logistic regression to evaluate potential informative missingness. Additional complete-case analyses yielded comparable discrimination estimates, indicating that the absence of incremental predictive improvement was not attributable to missing hormone measurements.

Because thyroid hormone measurements were obtained early (within 72 h) and DCI typically develops later during the clinical course, DCI was modeled as a confounder reflecting overall disease severity rather than as a mediator. DCI was defined according to the consensus criteria [31] as the occurrence of new focal neurological impairment or a decrease in Glasgow Coma Scale (GCS) score lasting for at least one hour, not attributable to other causes, and/or the appearance of new cerebral infarction on imaging not present on admission scans.

Continuous variables were summarized using means with standard deviations or medians with interquartile ranges, depending on their distribution, while categorical variables were presented as counts and percentages. Associations with 30-day in-hospital mortality were evaluated using univariable and multivariable logistic regression models adjusted for age, sex, APACHE II score, WFNS grade, Fisher grade, and treatment modality (endovascular coiling). Because DCI represents a post-admission complication, it was not included in the primary prognostic models and was evaluated only in secondary analyses.

Time-to-death analyses were performed using Cox proportional hazards models. Effect estimates were reported as odds ratios or hazard ratios with 95% confidence intervals. Model diagnostics and assumption checks were conducted, including assessment of proportional hazards using Schoenfeld residuals, with no significant violations detected. Linearity of continuous predictors of interest was evaluated using restricted cubic splines. A two-sided *p* value < 0.05 was considered statistically significant. Logistic regression was used to evaluate 30-day mortality as a binary endpoint, whereas Cox proportional hazards models assessed the effect of predictors on time-to-death; thus, OR and HR estimates reflect complementary but not directly interchangeable effect measures. OR and HR estimates are not numerically interchangeable because logistic regression models the cumulative 30-day risk, whereas Cox models account for timing of death and censoring; concordance of direction and magnitude was considered evidence of robustness rather than expected equality. The association between fT3 availability and outcome was formally evaluated to assess potential informative missingness.

All statistical analyses and data visualizations were performed using R statistical software (R Foundation for Statistical Computing, Vienna, Austria), version 4.5.2, within the RStudio integrated development environment (version 2025.09.2).

## 3. Results

### 3.1. Assessment of Missing fT3 Measurements

Patients without available fT3 measurements did not differ significantly in baseline severity (age, APACHE II, WFNS, or Fisher grade) from those with available measurements. Although crude mortality was numerically higher in this group, the difference was not statistically significant and was not independently associated with outcome after adjustment for clinical severity.

### 3.2. Early Thyroid Hormone Alterations

**Figure 3:**
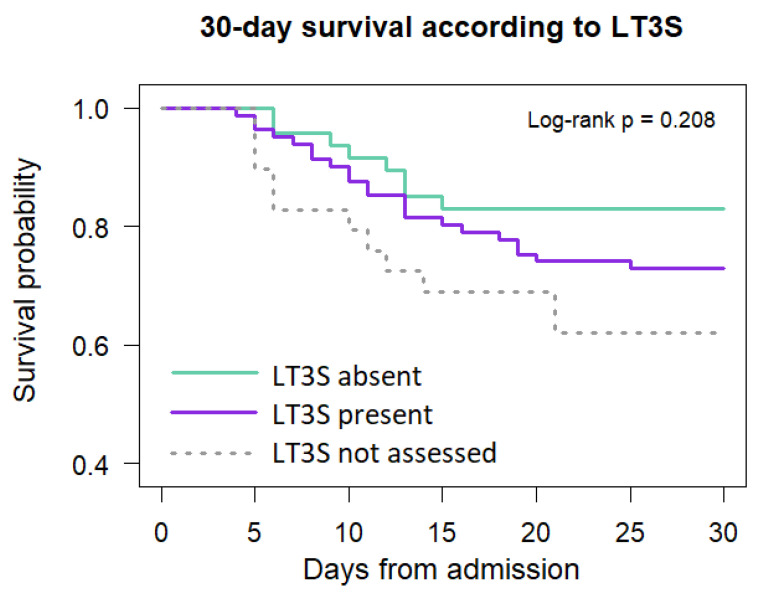
Kaplan–Meier survival curves showing 30-day survival in patients with and without low tri-iodothyronine syndrome (LT3S). Survival probabilities are shown from hospital admission to day 30. Although patients with LT3S demonstrated a numerically lower survival compared with those without LT3S, the difference did not reach statistical significance (log-rank test, *p* = 0.208). Patients without available hormonal measurements (LT3S not assessed) are shown for descriptive comparison only and were not included in inferential analyses.

Early peripheral thyroid hormone alterations were frequently observed in the acute phase of aSAH. Patients meeting criteria for LT3S tended to present with greater neurological severity and higher 30-day mortality; however, dichotomous LT3S classification did not retain independent prognostic significance in univariable or multivariable analyses, as shown in Figure 3.

Lower FT3_level values were more pronounced in patients treated with endovascular coiling compared with those undergoing surgical clipping, although treatment modality itself was not associated with early mortality. Importantly, no significant correlations were observed between raw fT3 deficit and standard inflammatory markers.

### 3.3. Peripheral fT3 and Central TSH Regulation

**Figure 4:**
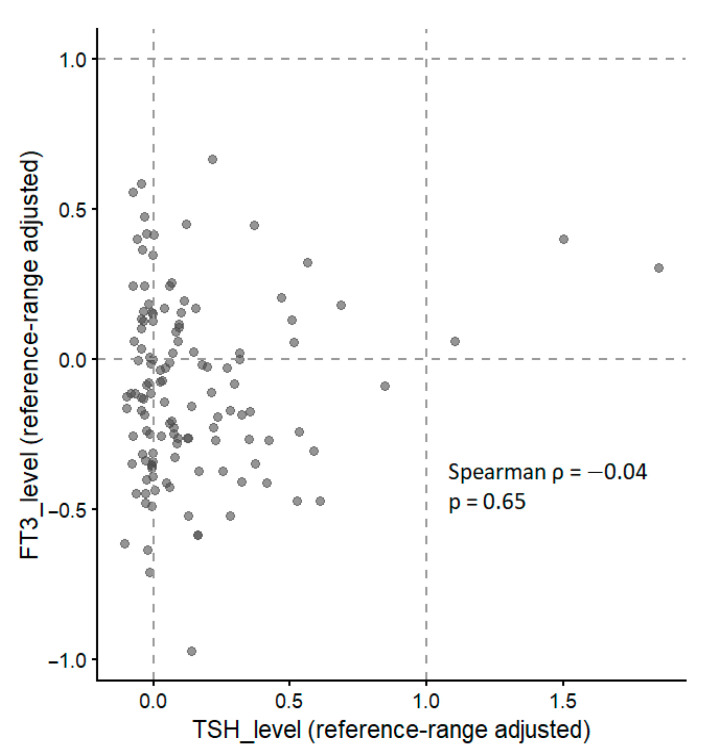
Dissociation between peripheral free tri-iodothyronine (fT3) availability and central thyroid-stimulating hormone (TSH) regulation. Scatter plot showing reference-range–adjusted FT3_level versus TSH_level measured within 72 h of admission. No significant correlation was observed (Spearman ρ = −0.04, *p* = 0.65). The dashed vertical and horizontal lines mark the limits of the laboratory reference ranges.

As seen in Figure 4, reference-adjusted FT3_level was not correlated with TSH_level (Spearman ρ = −0.04, *p* = 0.65), supporting the concept of NTIS characterized by impaired peripheral thyroid hormone metabolism independent of pituitary regulation. TSH_level did not differ across FT3_level strata and was not associated with 30-day mortality in univariable or multivariable analyses. No interaction between FT3_level and TSH_level was observed in models predicting early mortality.

### 3.4. Association Between fT3 Deficiency and Early Mortality

**Figure 5:**
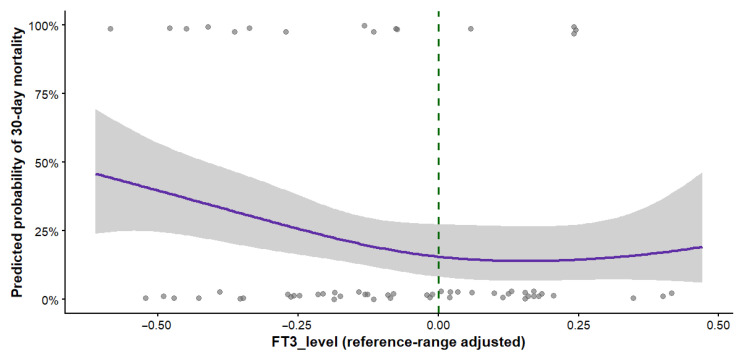
Association between reference-range–adjusted FT3_level and 30-day mortality in patients with aneurysmal subarachnoid hemorrhage. The solid line represents the predicted probability of 30-day mortality from a logistic regression model with a restricted cubic spline for FT3_level, with the shaded area indicating the 95% confidence interval. Wider confidence intervals at the extremes reflect limited data density and should be interpreted cautiously. Points represent individual patients. The dashed vertical line marks the lower limit of the laboratory reference range (FT3_level = 0). The association remained statistically significant after multivariable adjustment; predicted probabilities are derived from the multivariable logistic model; time-to-event effect estimates are presented in the Cox analysis.

Higher FT3_level was independently associated with decreased 30-day mortality and longer survival time in Cox proportional hazards models adjusted for age, sex, WFNS grade at admission, and APACHE II score. This association remained significant after inclusion of endovascular coiling in the model (adjusted HR 0.18, 95% CI 0.05–0.68; *p* = 0.011). After clinically interpretable rescaling, each 0.1-unit increase in FT3_level was associated with a 16% decrease in the hazard of death (adjusted HR 0.84, 95% CI 0.74–0.96; *p* = 0.011). Data are shown in Figure 5.

In logistic regression evaluating 30-day mortality as a binary outcome, each 0.1 increase in FT3_level was associated with an 18% lower odds of death (adjusted OR 0.82, 95% CI 0.69–0.97). The concordance between OR and HR estimates supports the robustness of the association across complementary analytical frameworks.

Lower FT3_level values were consistently associated with increased mortality risk across both logistic and time-to-event models.

**Figure 6:**
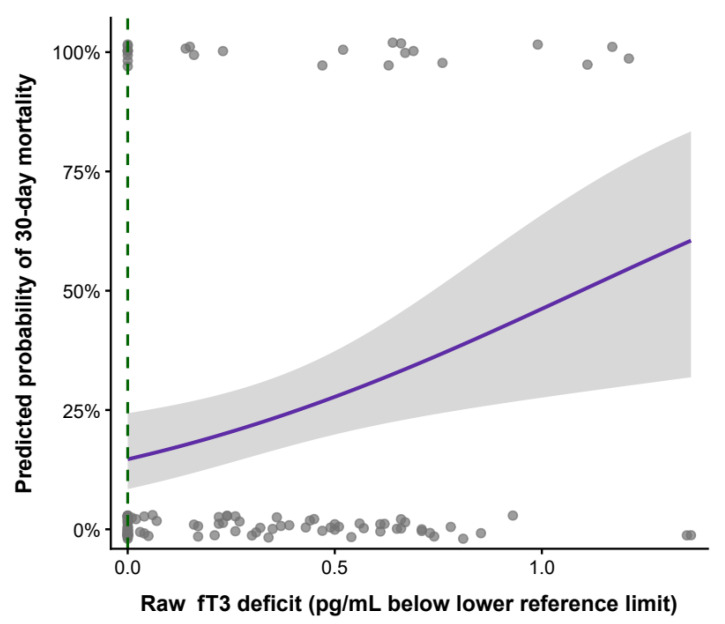
Association between raw free triiodothyronine (fT3) deficit and predicted 30-day mortality in patients with aneurysmal subarachnoid hemorrhage. Raw fT3 deficit was calculated as the difference between the lower laboratory reference limit and the measured fT3 concentration. Grey points represent individual patients, with values of 0 indicating survival and 1 indicating death within 30 days. The solid line shows the predicted probability of 30-day mortality derived from a logistic regression model with a restricted cubic spline for raw fT3 deficit, and the shaded areas represents the 95% confidence interval. The dashed vertical line marks the lower limit of the laboratory reference range (fT3 deficit = 0). The x-axis is truncated at zero for visualization purposes. Statistical analyses were performed across the full range of values, p for trend = 0.027.

Similarly, raw fT3 deficit exhibited a dose-dependent association with early mortality. Across the full range of values, each 0.1 pg/mL increase in raw fT3 deficit was associated with an approximately 8% increase in the hazard of 30-day mortality (adjusted HR 1.08 per 0.1 pg/mL, 95% CI 1.01–1.17; *p* = 0.027). Model performance was comparable between raw and reference-range–adjusted fT3 metrics, with minimal differences in information criteria (ΔAIC < 2). Visualization in Figure 6.

Addition of FT3_level to the baseline model resulted in a small numerical increase in AUC from 0.81 to 0.83 (ΔAUC = 0.026); however, this improvement did not reach statistical significance (DeLong test *p* = 0.23). In a secondary exploratory model including DCI as a post-admission variable, DCI was not independently associated with 30-day mortality (adjusted OR 1.97, 95% CI 0.70–5.96), and inclusion of DCI did not materially alter the effect estimate for FT3_level.

### 3.5. Aneurysm Location and Treatment Modality

Aneurysm location was not significantly associated with FT3_level or raw fT3 deficit. Across anatomical subgroups, the anterior communicating artery/anterior communicating artery (ACA/ACoA) group exhibited the lowest FT3_level values and the greatest fT3 deficits; however, these differences did not reach statistical significance.

Endovascular coiling, compared with neurosurgical clipping, was associated with greater reference-adjusted fT3 deficiency (*p* < 0.05), yet treatment modality itself was not associated with mortality after adjustment for clinical severity. Neither treatment modality was independently associated with 30-day mortality or survival time.

### 3.6. Neurological Outcomes and Delayed Cerebral Ischemia

Neither FT3_level nor raw fT3 deficit was associated with the occurrence of DCI in univariable or multivariable analyses. FT3_level was not independently associated with neurological deficit at ICU discharge or early functional outcome. Neurological outcomes were primarily determined by age, baseline neurological severity, and DCI. DCI was independently associated with poor functional outcome at discharge. In multivariable logistic regression, patients with DCI had nearly a fourfold higher odds of poor outcome (GOS 1–3) compared with those without DCI (OR 3.71, 95% CI 1.65–8.86), after adjustment for age, APACHE II score, and Fisher grade. Fisher grade and aneurysm location were not predictive of DCI.

## 4. Discussion

To our knowledge, this study is among the first to directly compare binary LT3S classification with continuous, reference-range–adjusted fT3 metrics in aSAH.

### 4.1. Principal Findings and Comparison with Existing Literature

In patients without pre-existing thyroid or pituitary disease, early thyroid hormone alterations observed after aSAH were most consistent with NTIS. Reduced T3 concentrations have been repeatedly linked to adverse outcomes in critically ill and neurocritical care populations [15,16,17,18,19,20,21,22,24,32]. Meta-analyses support an overall association between NTIS and mortality, although effect sizes vary and significance often diminishes after adjustment for illness severity [18,19]. Binary LT3S classification was common, but it was not independently associated with early mortality. This lack of statistical significance may partly reflect reduced statistical power inherent to dichotomization of a biologically continuous variable, together with the limited number of outcome events. In contrast, continuous, reference-range–adjusted measures of fT3 deficiency in our cohort were independently associated with 30-day mortality and shorter survival time, even after adjustment for established neurological and systemic severity scores. Although FT3_level remained independently associated with mortality, the incremental improvement in discrimination beyond established clinical variables was modest and did not reach statistical significance, possibly due to limited sample size and event count. These findings suggest limited incremental discriminative value beyond established clinical predictors and indicate that FT3_level should be interpreted primarily as a marker of biological vulnerability rather than as a stand-alone predictive tool.

### 4.2. Dissociation Between Peripheral fT3 and Central TSH Regulation

The absence of correlation between FT3_level and TSH_level supports the concept of NTIS, in which reduced peripheral T3 availability occurs independently of pituitary TSH signaling [11,33]. This dissociation likely explains why quantitative measures of fT3 deficiency—but not TSH—carry prognostic information in acute aSAH. The lack of association between aneurysm location and LT3S further supports a systemic metabolic mechanism rather than direct anatomical disruption of hypothalamic–pituitary structures. Similar patterns have been reported in heterogeneous emergency populations, in which lower total T3 levels were independently associated with greater disease severity, suggesting that T3 depletion reflects a generalized metabolic response to acute systemic stress rather than disease-specific mechanisms [34].

### 4.3. Magnitude of fT3 Deficiency as a Marker of Metabolic Vulnerability

Both raw fT3 deficit and FT3_level demonstrated independent associations with early mortality. Importantly, fT3 deficiency in our cohort was not correlated with standard inflammatory markers. This dissociation indicates that reduced peripheral T3 availability is not solely explained by conventional inflammatory markers and may represent a distinct dimension of the systemic stress response in acute aSAH. While raw deficit reflects absolute hormone depletion, FT3_level offers greater interpretability and robustness across evolving laboratory reference ranges. The comparable performance of both metrics indicates that risk is driven by the magnitude of peripheral T3 deficiency itself rather than by the specific method of quantification.

### 4.4. Mitochondrial Dysfunction as a Unifying Mechanism

The following considerations are hypothesis-generating and intended to provide biological context rather than mechanistic inference. Aneurysmal SAH imposes profound systemic and cerebral metabolic stress, requiring preserved mitochondrial function and efficient energy utilization. Key cytokines (such as interleukin-6 and tumor necrosis factor-α) suppress peripheral type 1 deiodinase while upregulating type 3 deiodinase activity [11,13]. As a result, the conversion of thyroxine (T4) to active T3 is reduced, whereas the production of biologically inactive reverse T3 is increased [13,14]. Triiodothyronine plays a central role in regulating mitochondrial respiration, oxidative phosphorylation, adenosine triphosphate production, and neuronal repair processes [29,35,36,37]. Reduced T3 availability may therefore reflect impaired metabolic flexibility and diminished energetic reserve, rendering patients less capable of tolerating acute physiological stress [15,18,33].

### 4.5. Aneurysm Characteristics, Treatment Modality, and Thyroid Response

Although aneurysm location was not independently associated with fT3 deficiency, the consistent pattern of lower FT3_level values in ACA/ACoA aneurysms raises the possibility of location-related modulation of systemic metabolic stress rather than a direct anatomical effect.

Endovascular coiling, compared with neurosurgical clipping, was associated with greater peripheral fT3 deficiency despite its less invasive nature. This may reflect exposure to iodinated contrast agents, which can acutely inhibit thyroid hormone synthesis and peripheral T4-to-T3 conversion via the Wolff–Chaikoff effect [38], as well as cytokine-mediated suppression of deiodinase activity [11,13]. Surgical clipping, associated with greater physiological stress, was likewise linked to more pronounced fT3 deficiency. Importantly, neither treatment modality influenced early mortality, and inclusion of treatment variables did not attenuate the prognostic effect of FT3_level. These findings suggest that fT3 deficiency reflects individual metabolic vulnerability rather than the invasiveness of aneurysm occlusion strategy.

### 4.6. Dissociation Between Survival and Neurological Recovery

DCI remains one of the most devastating secondary complications of aSAH, exerting a dominant influence on early neurological outcome and functional recovery, independent of initial hemorrhage severity [31]. Notably, quantitative fT3 deficiency was not associated with the development of DCI, indicating that reduced peripheral T3 availability does not contribute directly to the pathophysiological mechanisms underlying DCI. Instead, fT3 deficiency appears to reflect a systemic metabolic vulnerability influencing survival rather than cerebrovascular complications per se. Although DCI is a major determinant of neurological deterioration, its lack of independent association with early mortality after adjustment suggests that systemic metabolic failure and primary injury severity may play a more dominant role in determining short-term survival.

Quantitative fT3 deficiency was independently associated with early mortality but not with early neurological status or functional outcome at discharge. Neurological recovery was primarily driven by age, baseline neurological severity, and DCI. This dissociation suggests that survival and neurological recovery represent partially independent biological dimensions in acute aSAH [18,24,29], with FT3_level capturing systemic metabolic resilience rather than cerebrovascular complications such as DCI.

### 4.7. Clinical and Translational Implications

Binary LT3S classification alone appears insufficient for prognostic stratification in aSAH [22,23]. In contrast, early quantitative assessment of fT3 deficiency using standardized, reference-adjusted metrics may help identify patients with heightened metabolic vulnerability who could warrant closer observational assessment in future prospective studies [29,35]. Incorporation of fT3 measures into multimodal prognostic models complements established neurological grading scales and aligns with contemporary biomarker development principles [22,23,33]. Routine early assessment of fT3 may be particularly useful in patients with discordant neurological and systemic severity profiles.

### 4.8. Limitations

This study has several limitations. Its retrospective single-center observational design may limit generalizability. Missing fT3 measurements occurred in a minority of patients and were not associated with baseline disease severity. While crude mortality appeared higher among patients without hormone assessment, this relationship was not independent after adjustment, suggesting that missingness primarily reflected logistical constraints of retrospective ICU practice rather than systematic bias. Nevertheless, the possibility of residual selection effects cannot be entirely excluded. Thyroid hormone measurements were obtained at a single early time point, which precluded assessment of temporal trajectories of NTIS [14,28]. Accordingly, FT3 values should be interpreted as markers of the biological response during the EBI phase rather than static indicators of baseline thyroid status. Because thyroid hormone alterations in critical illness evolve over time, we were unable to determine whether persistent versus transient reductions in fT3 carry different prognostic implications and therefore cannot distinguish pre-existing vulnerability from dynamic illness-related changes. Although reference-range normalization reduces systematic differences related to assay-specific intervals, it cannot fully account for all sources of analytical variability inherent to retrospective multi-assay data. Residual confounding by illness severity cannot be entirely excluded, as more severe systemic stress may simultaneously drive both fT3 depletion and mortality risk. Mechanistic interpretation is limited because reverse T3 was not available, preventing full biochemical characterization of NTIS and limiting pathophysiological interpretation of altered deiodinase activity [14]. The requirement for aneurysm occlusion within 48 h may have excluded patients who died prior to intervention or were deemed non-operable, potentially introducing survivorship bias. External validation in an independent cohort was not available. In addition, the extended inclusion period may reflect temporal changes in case mix and neurocritical care practice that cannot be fully controlled in a retrospective design. The modest and statistically non-significant improvement in discrimination suggests that fT3 should be interpreted primarily as a marker of biological vulnerability rather than as a tool for risk reclassification beyond established clinical scores.

## 5. Conclusions

Early reduction in fT3 is associated with clinically relevant metabolic disturbance in patients with aSAH. Continuous, reference-range–adjusted measures of fT3 deficiency—rather than binary LT3S classification—were independently associated with 30-day mortality after adjustment for established severity markers. Quantitative assessment of fT3 deficiency enhances early risk stratification and may support clinical decision-making in patients with aSAH.

## Figures and Tables

**Table 1:** Baseline characteristics were summarized for the entire cohort and stratified by LT3S status. Patients with missing fT3 measurements (n = 26) were excluded from group comparisons. * Data are presented as median [interquartile range] or n (%). ** *p*-Values refer to comparisons between the LT3S and without LT3S groups only. Patients without available fT3 measurements (LT3S not assessed) are presented descriptively and were not included in inferential comparisons. DCI—delayed cerebral ischemia; ICU—Intensive Care Unit; WFNS—World Federation of Neurosurgical Societies; GCS—Glasgow Coma Scale; GOS—Glasgow Outcome Scale; LT3S—low-triiodothyronine syndrome.

Characteristic	Whole Cohort n = 157 *	LT3S Present n = 81 *	LT3S Absent n = 50 *	LT3S Not Assessed n = 26 *	*p*-Value **
sex					0.7
female	100 (64%)	54 (67%)	31 (62%)	15 (58%)	
male	57 (36%)	27 (33%)	19 (38%)	11 (42%)	
age	56 (44–67)	57 (48–67)	52 (39–66)	59 (42–65)	0.255
APACHE II	17 (11–27)	17 (11–26)	19 (10–28)	17 (11–29)	0.612
GCS	11.0 (5.0–14.0)	12.0 (5.0–14.0)	10.0 (7.0–14.0)	13.0 (4.0–14.0)	0.824
Fisher grade					0.4
1	4 (2.5%)	1 (1%)	3 (6%)	0 (0%)	
2	25 (16%)	13 (16%)	6 (12%)	6 (23%)	
3	34 (22%)	20 (25%)	8 (16%)	6 (23%)	
4	94 (60%)	47 (58%)	33 (66%)	14 (54%)	
WFNS grade					0.7
1	29 (18%)	16 (20%)	9 (18%)	4 (15%)	
2	32 (20%)	13 (16%)	11 (22%)	8 (31%)	
3	12 (7.6%)	8 (10%)	2 (4%)	2 (8%)	
4	33 (21%)	17 (21%)	13 (26%)	3 (12%)	
5	51 (32%)	27 (33%)	15 (30%)	9 (35%)	
Treatment modality					0.001
Endovascular coiling	117 (74.5%)	52 (64%)	46 (92%)	16 (62%)	
Surgical clipping	40 (25.5%)	29 (36%)	4 (8%)	7 (27%)	
hypertension	73 (46%)	45 (56%)	16 (32%)	12 (46%)	0.034
smoking	85 (54%)	41 (51%)	31 (62%)	13 (50%)	0.4
DCI	88 (56%)	49 (60%)	29 (58%)	10 (38%)	0.15
Functional Outcome					
Poor outcome (GOS 1–3)	88 (56%)	45 (56%)	31 (62%)	12 (46%)	
Good outcome (GOS 4–5)	69 (44%)	36 (44%)	19 (38%)	14 (54%)	
ICU length of stay, days	10 (7–18)	11 (7–18)	11 (7–19)	8 (5–13)	0.922
in hospital mortality					0.4
alive at day 30	116 (74%)	59 (73%)	40 (80%)	17 (65%)	
dead at day 30	41 (26%)	22 (27%)	10 (20%)	9 (35%)	
fT3		1.72 (1.49–2.03)	2.12 (1.94–2.64)		<0.001
raw fT3 deficit		0.47 (0.24–0.67)	−0.34 (−0.66–−0.18)		<0.001
FT3_level		−0.26 (−0.39–−0.13)	0.17 (0.09–0.35)		<0.001
TSH_level		0.05 (−0.02–0.21)	0.07 (−0.03–0.31)	0.15 (0.02–0.35)	0.015

## Data Availability

The data presented in this study are not publicly available due to ethical and privacy restrictions, as they contain potentially identifiable clinical information. Anonymized data may be made available from the corresponding author upon request.

## Abbreviations

The following abbreviations are used in this manuscript:

aSAH	aneurysmal subarachnoid hemorrhage
NTIS	non-thyroidal illness syndrome
LT3S	low-triiodothyronine syndrome
fT3	free triiodothyronine
fT4	free thyroxine
TSH	thyroid-stimulating hormone
WFNS	World Federation of Neurosurgical Societies
GCS	Glasgow Coma Scale
GOS	Glasgow Outcome Scale
EBI	early brain injury
DCI	delayed cerebral ischemia
T3	triiodothyronine
T4	thyroxine
CT	computed tomography
CTA	computed tomography angiography
DSA	digital subtraction angiography
ACA	anterior cerebral artery
ACoA	anterior communicating artery
ICU	intensive care unit
LRL	lower reference limit
URL	upper reference limit
